# Development and implementation of a cell-based assay to discover agonists of the nuclear receptor REV-ERBα

**DOI:** 10.14440/jbm.2018.244

**Published:** 2018-06-25

**Authors:** Yuliya Hering, Alexandre Berthier, Helene Duez, Philippe Lefebvre, Benoit Deprez, Philip Gribbon, Markus Wolf, Jeanette Reinshagen, Francoise Halley, Juliane Hannemann, Rainer Böger, Bart Staels, Sheraz Gul

**Affiliations:** 1 Fraunhofer Institute for Molecular Biology and Applied Ecology, ScreeningPort, Schnackenburgallee 114, D-22525 Hamburg, Germany; 2 University of Lille-EGID, CHU, Institut Pasteur de Lille, INSERM UMR 1011, 1 rue du Professeur Calmette, BP245, 59019 Lille, France; 3 University Lille Nord de France, INSERM, Institut Pasteur de Lille, U1177, Drugs and Molecules for Living Systems, F-59000 Lille, France; 4 Institute of Clinical Pharmacology and Toxicology, University Medical Center Hamburg-Eppendorf, Martinistraße 52, D-20246 Hamburg, Germany

**Keywords:** nuclear receptor, REV-ERBα, luciferase reporter, assay development, drug discovery, high throughput screening

## Abstract

The nuclear receptors are transcription factors involved in the regulation of a variety of physiological processes whose activity can be modulated by binding to relevant small molecule ligands. Their dysfunction has been shown to play a role in disease states such as diabetes, cancer, inflammatory diseases, and hormonal resistance ailments, which makes them interesting targets for drug discovery. The nuclear receptor REV-ERBα is involved in regulating the circadian rhythm and metabolism. Its natural ligand is heme and there is significant interest in identifying novel synthetic modulators to serve as tools to characterize its function and to serve as drugs in treating metabolic disorders. To do so, we established a mammalian cell-based two-hybrid assay system capable of measuring the interaction between REV-ERBα and its co-repressor, nuclear co-repressor 1. This assay was validated to industry standard criteria and was used to screen a subset of the LOPAC^®1280^ library and 29568 compounds from a diverse compound library. Profiling of the primary hits in a panel of counter and selectivity assays confirmed that REV-ERBα activity can be modulated pharmacologically and chemical scaffolds have been identified for optimization.

## INTRODUCTION

Nuclear receptors (NRs) are members of a superfamily of ligand-regulated transcription factors that mediate gene transcription in response to endogenous lipophilic small molecules such as steroid hormones, thyroid hormones, lipids, fatty acids, and retinoic acids [1]. Since members of this superfamily are involved in many physiological processes and play an important role in humans, they are a major target class for therapeutic intervention to treat human diseases. NRs modulate the transcription of their target genes in most instances as a consequence of direct binding at specific DNA sequences in the promotor region of their target genes, also referred to as response elements (RE) [2]. NRs bind specific DNA elements in the regulatory regions of genes *via* a highly conserved DNA binding domain (DBD). Further, they bind specific ligands *via* the ligand binding domain (LBD) that contains a hydrophobic pocket. The binding of ligands in the pocket induces conformational changes in the receptor that affects the recruitment of co-regulatory molecules (cofactors) that stimulate (co-activators) or repress (co-repressors) transcription of the target gene [3]. The NRs with unknown ligands are referred to as orphan receptors of the superfamily [4]. To date, 48 NRs are known; however, physiological ligands have been identified for only around half of them [5,6].

REV-ERBα and REV-ERBβ are members of the nuclear hormone receptor superfamily which are expressed throughout the body [3]. Both receptors have a circadian pattern of expression that is essential to circadian and metabolic regulations. REV-ERBα recognizes two classes of DNA response elements and can bind to DNA as a monomer, to a (A/G) GGTCA half site with a 5’ AT-rich extension or as a homodimer to a direct repeat 2 element (AGGTCA sequence with a 2-bp spacer) [3].

**Figure 1.**
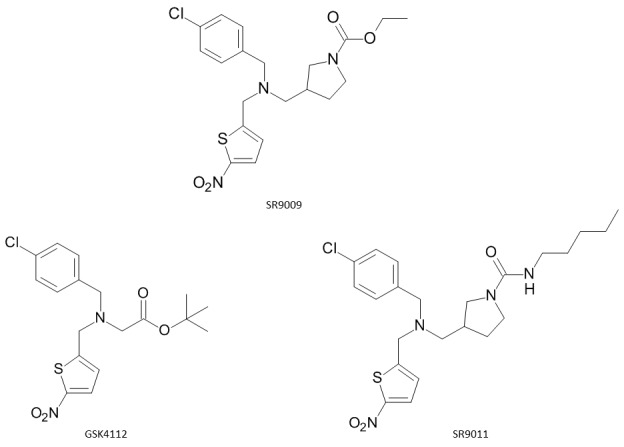
Structural formulae of the synthetic ligands GSK4112, SR9011 and SR9009.

Experimental and clinical research has indicated that circadian disruption caused in humans by shift work, for example, causes metabolic disturbances [7-9]. Genetic ablation of the core CLOCK genes in mice leads to a range of abnormal metabolic phenotypes including obesity, dyslipidemia, and glucose intolerance, indicating that circadian rhythms are linked to the regulation of metabolism [10,11]. Therefore, synthetic ligands that modulate REV-ERBα activity may hold utility in treatment of several metabolic disorders such as obesity and type 2 diabetes [12]. Heme binds reversibly to the LBD of REV-ERBα with an affinity of 2–4 μM and thereby modulates the ability of the receptor to recruit the co-repressor NCoR [3]. This finding has led to the search for synthetic REV-ERBα ligands that pharmacologically target the circadian rhythm and may hold utility in the treatment of sleep disorders as well as metabolic diseases [13]. The first synthetic REV-ERBα ligand to be identified was 1,1-dimethyl-ethyl-N-[(4-chlorophenyl) methyl]-N-[(5-nitro-2-thienyl) methyl] glycinate (originally named SR6452 and subsequently GSK4112) shown in **Figure 1.** The compound was identified from a fluorescence resonance energy transfer (FRET) biochemical screen in which it was shown to increase the interaction between REV-ERBα and NCoR in a concentration-dependent manner [14]. This non-porphyrin synthetic ligand for REV-ERBα mimics the action of heme and behaves as an agonist. In cell-based assays, GSK4112 was able to reset circadian rhythms [15,16]. However, it displays low systemic exposure and weak efficiency as a REV-ERBα agonist. It is therefore not suitable for *in-vivo* experiments [17,18]. Based on the GSK4112 scaffold, two other synthetic REV-ERB ligands, SR9009 and SR9011, have been synthesized (**Fig. 1**) with improved potency, efficacy and pharmacokinetic properties [19]. These compounds are synthetic agonists of REV-ERBα and REV-ERBβ and of potential use to treat circadian and metabolic disorders [17,19]. In reporter gene luciferase assays, SR9009 and SR011 were shown to be 3–4-fold more potent than GSK4112 [20].

**Figure 2.**
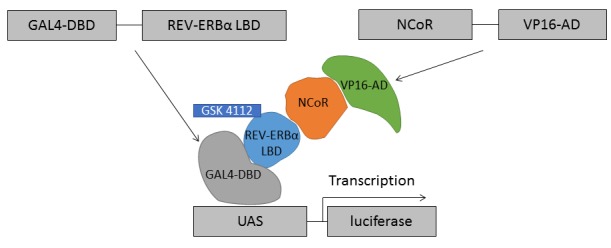
Schematic depiction of the mammalian cell-based two-hybrid system measuring the interaction between nuclear receptor REV-ERBα and corepressor NCoR. Plasmids were co-transfected into HEK-293T cells, and then the Renilla and firefly luciferase signals were quantified using the Dual-Glo^®^ Luciferase System 24 h post-transfection. In the mammalian cell-based two-hybrid system, the DNA-binding domain is co-expressed with human REV-ERBα and the transcription activation domain is co-expressed with NCoR. The interaction of REV-ERBα and NCoR leads to transcription of firefly luciferase. GSK4112 is a REV-ERBα agonist binding to the heme binding site resulting in recruitment of the co-repressor NCoR.

In the study reported herein, we describe the development of a screening compatible mammalian cell-based two-hybrid assay (**Fig. 2**) that offers the ability to identify REV-ERBα agonists. The assay made use of HEK-293T cells that underwent transfection with plasmids including the key target under investigation, namely REV-ERBα. The assay was designed to have a dual luminescence readout (firefly and Renilla luciferase enzyme activities), as these enzymes allow to construct good dual-reporter assays due to their differences in substrate requirements and light output. The assay readout was normalized using the Renilla luciferase signal as this takes into account any changes in signal due to target-unspecific effects such as growth arrest, cytotoxicity, and protein expression manipulation. A number of assay parameters were investigated so as to ensure it was miniaturized into 384-well microtiter plate format, including optimization of plasmid/DNA, cell number, cell culture media components, order of addition of reagents, DMSO tolerance, reproducibility, Z' and pharmacology using the available synthetic ligands (GSK4112, SR9009 and SR011). The validated assay was subsequently employed in a high throughput screen (HTS) campaign to screen a subset of the Library of Pharmacologically Active Compounds (LOPAC^®1280^, Sigma-Aldrich) as well as 29568 diverse compounds from Enamine (Kiev, Ukraine). The smaller screen led to the identification of chelidamic acid as a validated hit and the larger screen led to the identification of 63 hits of which 48 were confirmed in dose-response studies to be REV-ERBα agonists. The confirmed hits were evaluated in secondary assays and these scaffolds can be deemed as chemical starting points for REV-ERBα agonist drug discovery.

## MATERIALS AND METHODS

### Materials

Buffers, cell culture media and serum were of the highest quality and purchased from Sigma-Aldrich unless otherwise stated. The Library of Pharmacologically Active Compounds (LOPAC^®1280^) was purchased from Sigma-Aldrich and 29568 diverse compounds were purchased from Enamine (Kiev, Ukraine). All compounds were dissolved in DMSO (1 mM solutions LOPAC^®1280^/2 mM Enamine) and stored at **−**20°C in 384-well Labcyte microtiter plates.

### Plasmids

Expression vector pGal4-Rev-erbα was created by cloning the human REV-ERBα DEF regions in a pGal4 plasmid (pM from Clonetech) which contains the Gal4 DBD sequence. The pVP16-NCoR vector corresponds to NCoR interaction domain (ID1, ID2, and ID3) sequences fused to VP16 transactivation domain cloned into a pcDNA expression vector (Life Technologies, CA, USA). The reporter vector contains luciferase gene under control of Gal4 response element (UAS) and minimal TK promoter sequences [21] and the normalization vector pCMV-Ren was from Promega Inc., USA. For the secondary screening assay, the pGAL4-hCAR1 expression vector was created by cloning full length human CAR1 (NM_005122.4) sequence in the Gal4 plasmid. The pGal4-hPXR expression vector contains DEF regions of human PXR cloned into the Gal4 plasmid [22]. All sequences are available upon request.

After heat shock of competent *E. coli* XL1 blue cells (Agilent Technologies, CA, USA), isolation and purification of plasmids were performed using EndoFree (endotoxin free) plasmid purification kit (QIAGEN, Hilden, Germany) as instructed by the manufacturer. Restriction site analysis was used to confirm each plasmid preparation.

### Development and validation of the REV-ERBα mammalian cell-based two-hybrid reporter assay

HEK-293T cells were obtained from DSMZ (German Collection of Microorganisms and Cell Cultures, Braunschweig, Germany) and grown in DMEM (Lonza Inc., Walkersville, USA) supplemented with 10% v/v FBS, 100 μg/ml streptomycin and 100 U/ml penicillin G (Biochrom AG, Berlin). The cells were grown at 37°C in a humidified atmosphere in the presence of 5% CO_2_ and only those with passage number of 4–24 were used for the transfection experiments. Upon growth of the HEK-293T cells to 80%–90% confluency, the cells were washed with 10 ml pre-warmed PBS and detached by incubation for 2–4 min with 3 ml of trypsin/EDTA (0.05%/0.02% v/v) at 37°C in the presence of 5% CO_2_. Trypsin was inactivated by addition of 7 ml culture medium. Cells were then transferred into culture flask containing fresh pre-warmed cell culture medium and split to a 1:10 ratio.

The assay made use of cells cultured as described above which were subsequent to harvesting in starvation conditions (medium supplemented with DMEM supplemented with 1% DCC FBS, 100 μg/ml streptomycin and 100 U/ml penicillin G) and then undergoing bulk transfection by the addition of 0.1 µl jetPEI/0.05 µg DNA (0.037 µg p-UAS-TK-Luc, 0.005 µg Gal4 (NR1D1 or empty), 0.005 µg pNCOR-VP16 and 0.003 µg p-Ren) per 20 µl of cells at room temperature for 45 min. This suspension was seeded into 384-well assay plates (20 μl/well) (CellStar, white, PS Greiner Bio-One GmbH) and grown at 37°C in a humidified atmosphere in the presence of 5% CO_2_ for 6 h. Subsequently, 100 µM GSK4112 agonist (positive control), test compounds (1 mM stock solutions in 100% DMSO v/v to give 4 µM final assay concentration), or DMSO only (negative control) were added to each well using the Labcyte Echo 550 (Labcyte, Sunnyvale, CA) acoustic dispenser and incubated for further 18 h (37°C in a humidified atmosphere in the presence of 5% CO_2_). Following this, the interaction between REV-ERBα and NCoR was investigated using Dual-Glo^®^ Luciferase System using the instructions provided by the manufacturer (Promega Inc., USA). Plate handling was performed using a Cell Explorer HTS platform (PerkinElmer, Waltham, MA), liquid handling was performed using the Multidrop (Thermo, Waltham, MA) and luminescence measurements were made using an EnVision Multilabel Reader (PerkinElmer, Waltham, MA).

### Counter and secondary screening

For counter and secondary screening harvested HEK-293T cells (1 × 10^6^) were mixed with a mixture of plasmids for counter (p-uas-tk-luc 1 µg, pCMVRen 0.1 µg, pGal4-Rev-erbα 0.5 µg and pVP16-NCoR 0.5 µg) and secondary screening assays (p-uas-tk-luc 1 µg, pCMVRen 0.1 µg, pGal4-hPXR or pGal4-hCAR1 0.5 µg). The jetPEI transfection reagent solution was prepared just before use (5 μl/well). The jetPEI/DNA mix was incubated for complex formation at room temperature for 20 min and added to the cell suspension. After 24 h incubation, transfected cells were seeded on 96-well assay plates (~50000 cells/well) with 10% DCC-FBS. The following day, cells were treated for 24 h with compounds (at 10 µM) or DMSO (0.1% v/v) in phenol red free DMEM with 1% DCC-FBS Renilla and firefly luciferase activities were quantified using Dual-Glo^®^ Luciferase System using the instructions provided by the manufacturer and Victor Light luminometer (PerkinElmer, Waltham, MA). For Gal4-hCAR assay, cells were co-treated with an inverse agonist of hCAR1 (PK11195, 10 µM) in order to prevent the constitutive activity of the construct.

### Data analysis

In order to normalize the raw screening data, the measured relative luminesce units (RLU) generated by the converted firefly luciferase were divided by the measured Renilla RLU to calculate % Effect within ActivityBase XE (ID Business Solutions Ltd, UK) and visualized using Spotfire^®^ (PerkinElmer Inc., USA). For dose-response experiments, the IC_50_ values were calculated by fitting the duplicate data to the four-parameter logistic equation in GraphPad Prism (version 5.02, GraphPad Software, Inc., USA) or ActivityBase XE.

## RESULTS

### Optimization of the REV-ERBα mammalian cell-based two-hybrid assay

**Figure 3.**
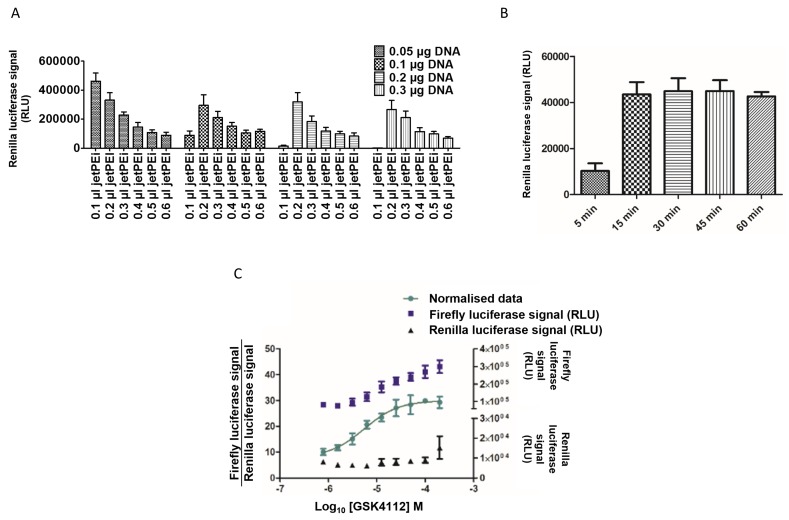
Characterization of the REV-ERBα mammalian cell-based two-hybrid assay. **A.** Determination of the appropriate DNA to transfection reagent ratio (N/P) in the REV-ERBα mammalian cell-based two-hybrid assay. HEK-293T cells (10000 cells/well) were transiently transfected with different amount of DNA (0.05–0.3 μg/well) in combination with different jetPEI concentrations (0.1–0.6 μl/well). The Renilla luciferase signal was measured 24 h post transfection using the Dual-Glo^®^ Luciferase System. **B.** Effect of complex formation incubation time on transfection efficiency with jetPEI. HEK-293T cells were transfected in suspension with the plasmids in batch mode. The jetPEI reagent was added to DNA sample and the complex was incubated for 5, 15, 30, 45 and 60 min. The Renilla luciferase signal was measured 24 h post transfection using the Dual-Glo^®^ Luciferase System. **C.** Dose-response of the GSK4112 agonist in the assay. HEK-293T cells were transiently transfected with four plasmids for 8 h followed by stimulation by GSK4112 for 16 h. The data were normalized by ratio (firefly luciferase signal/Renilla luciferase signal) and fitted using GraphPad Prism. Calculation of GSK4112 EC_50_ (5.5 µM) and Hill slope (1.21) was performed using GraphPad Prism. Results represent normalized mean values per well (triplicates). Error bars show the STD.

The development of a cell-based assay for REV-ERBα involved the optimization of many variables which were undertaken in a systematic manner to yield a reproducible screening compatible assay. The optimal jetPEI/DNA known as the N/P ratio (the ratios of moles of the amine groups of cationic polymers to those of the phosphate ones of DNA), is an important factor that affects the transfection efficiency and viability of cells. In order to obtain a positively charged complex it is recommended by the manufacturer to use a N/P ratio of > 3. In the case of this assay, 0.1 µl jetPEI was shown to yield the highest signal (**Fig. 3A**), with an incubation time with DNA of 45 min that would facilitate scheduling of a screening campaign (**Fig. 3B**). The optimal incubation times for the transfection and subsequent addition of compounds were shown to be 6 h and 18 h respectively as these conditions yielded IC_50_ value of 5.5 µM for the reference compound GSK4112, which is comparable to those reported in the literature (**Fig. 3C**). The two other known REV-ERBα ligands, namely SR9009 and SR9011 with improved efficacy and pharmacokinetic properties relative to GSK4112 were also characterized in the assay in order to ascertain whether these could be more appropriate positive controls in the screening campaign. Both SR9009 and SR9011 were associated with a cytotoxic effect at high concentrations. Therefore, these were deemed not suitable as a potential positive control compound in any screening campaign that was intended to be performed. Therefore, as the GSK4112 agonist led to the highest increase in firefly luciferase activity (with no effect upon the Renilla signal) and thus the highest increase in the interaction between REV-ERBα and NCoR, this compound was considered to be a potential positive control in subsequent assays.

### Cytotoxic effect of DMSO, GSK4112, SR9009 and SR9011

The DMSO tolerance of the assay was investigated between 0.1%–5% v/v DMSO by addition to non-transfected HEK-293T cells (10000 cells/well) and incubated for 24 h. The measurement of the cell viability was determined using the CellTiter-Glo^®^ assay which measures cellular ATP concentrations. DMSO exhibited a slight cytotoxic effect upon the HEK-293T cells at concentrations > 1% v/v DMSO. The cytotoxic effect of GSK4112 was determined by its incubation with non-transfected cells at concentrations (up to 200 μM) using the CellTiter-Glo^®^ assay performed after 18 h incubation, and no cytotoxic effect was observed. It has been recently shown that both SR9009 and 9011 REV-ERB agonists are potential anticancer drugs which presented dose-dependent toxic effects on several cancer cell lines [23]. In addition, we have observed that these molecules were associated with a decrease in Renilla luciferase signal at concentrations as low as 5 µM. Therefore, these compounds were not used as reference compounds.

### Screening of the REV-ERBα mammalian cell-based two-hybrid assay against two compound libraries

**Figure 4.**
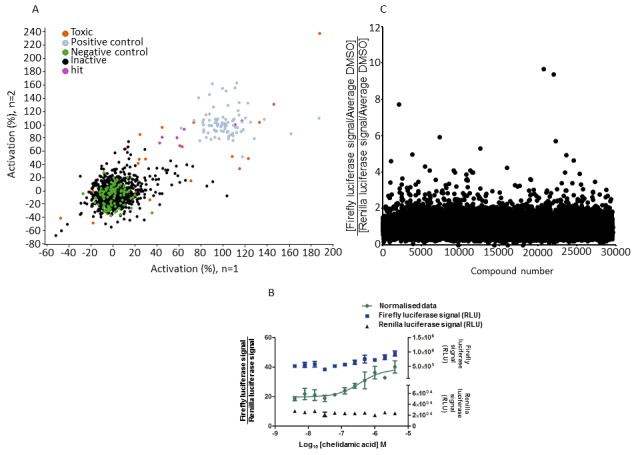
Screening of a subset of the LOPAC®1280 and diverse Enamine compound libraries. **A.** Correlation of activation (%) from two independent experiments by compounds from the LOPAC®1280 library. The firefly and Renilla luciferase signals were measured using the Dual-Glo® Luciferase System. The data were normalized by ratio (firefly luciferase signal/Renilla luciferase signal). The average activation (%) based upon the ratio firefly luciferase signal/Renilla luciferase signal measured on day 1 and 2 are plotted on the y and x-axes respectively [green: negative control (0.5% v/v DMSO); light blue: positive control (100 μM GSK4112); black: test compounds (inactive compounds); purple: hits (active compounds); orange: toxic compounds]. **B.** Dose-response of chelidamic acid in the assay. The data were normalized by ratio (firefly luciferase signal/Renilla luciferase signal) and fitted using GraphPad Prism. Results represent normalized mean values per well (triplicates). Error bars show the STD. Calculation of chelidamic acid EC50 (0.36 µM) and Hill slope (1.23) was performed using GraphPad Prism. **C.** Scatter-plot for the screening of the Enamine compound library against the REV-ERBα mammalian cell-based two-hybrid assay.

In order to verify that the assay performed acceptably under automated conditions, a training set of the Library of Pharmacologically Active Compounds (LOPAC^®1280^) was screened. The library was chosen because it is commonly employed to validate new drug discovery assays. A total of 960 compounds of the LOPAC^®1280^ library at a final assay concentration of 4 μM were screened in duplicate on different days. The data from each plate was normalized using the positive control GSK4112 (final concentration 100 μM in a complete column) and negative control (0.4% v/v DMSO in a complete column). The Z' [24] for each assay plate were 0.5, 0.6, 0.6, 0.6, 0.4 and 0.6, giving an average Z' of 0.55 and a good correlation between the two data sets obtained from two days could be observed indicating that the established mammalian cell-based two-hybrid assay provided reliable results (**Fig. 4A**). Compounds which showed > 50% activation based on the normalized data and < 50% effect of Renilla signal relative to DMSO, highlighted in **Figure 4A**, were defined as hits. The invalidation of cytotoxic compounds based on the Renilla signal being < 50% Renilla signal relative to DMSO resulted in the identification of 10 non-cytotoxic hits. Chelidamic acid showed activity against REV-ERBα in a concentration dependent manner and was associated with an EC_50_ of 0.36 μM (**Fig. 4B**). No increase in the firefly luciferase expression could be detected in the transfected cells with an empty vector (Gal4) instead of REV-ERBα plasmid (control transfection). This suggested that chelidamic acid binds specifically to the LBD site of REV-ERBα receptor leading to increased interaction between the co-expressed proteins REV-ERBα and NCoR resulting in an increase of firefly luciferase expression. Hence, chelidamic acid can be considered as a validated hit.

A large-scale screen was subsequently performed against 29568 diverse compounds from the Enamine compound library (**Fig. 4C**). In this case, the positive control, GSK4112 showed throughout the screening campaign a high day-to-day, plate-to-plate and also intra-plate variability and therefore could not be used as reference for 100% activation control and Z' calculation. Instead the quality control was solely based on robustness of baseline luminescence intensities and plates were considered invalid if absolute counts were significantly increased or decreased relative to the general observations caused by for example variability in transfection efficiency, and patterns caused by technical issues such as mis-dispense and evaporation effects. The raw firefly and Renilla luciferase luminescence intensity of all valid plates was normalized to the plate average DMSO signal which was set to 1. This yielded a homogenous DMSO and mean compound response throughout the entire screen. Agonists were characterized by compounds that yielded (1) relative Renilla signal within 2 σ of the average DMSO relative Renilla signal and (2) relative firefly signal positively exceeding the average DMSO relative firefly signal by > 10 σ. Using this criteria, 63 hits (hit-rate of ~0.2%) were selected of which 15 were antagonist hits and 48 were agonist hits. In total, the screening campaign made use of 84 plates that were screened over a period of 11 d in batches ranging from 5–18 plates and performed with similar quality as the LOPAC^®1280^ screen with the hits distributed randomly in each screening batch.

### Confirmation and secondary assays

**Figure 5.**
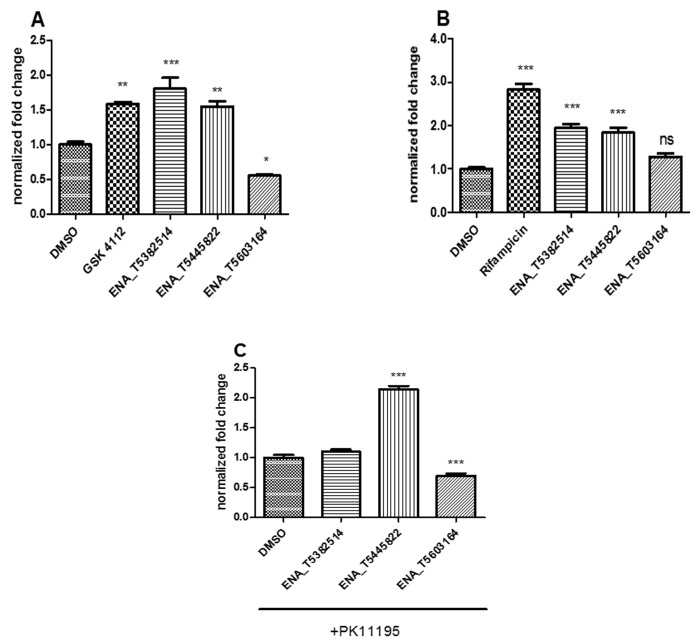
Secondary screening of selected hits from diverse Enamine compound screen. **A.** Counter screen using mammalian two hybrid assay. HEK-293T cell were transfected with Gal4-REV-ERBα and VP16-NCoR as described in Figure 1. **B** and **C.** Secondary screen assay using a mammalian single hybrid assay (without any VP16 construct). **B.** Cells were transfected with Gal4-hPXR. **C.** Cells were transfected with Gal4-hCAR. Transfected cells were treated for 24 h with DMSO (0.1% v/v) or 10 µM of selected compounds and luciferase assay were performed as described above. Results are expressed as mean ± SEM and statistical significance were assessed using GraphPad prism software by a 1-Way ANOVA followed by a Tuckey post-hoc test, *P < 0.05, **P < 0.01, ***P < 0.001.

The activities for three compounds (ENA_T5382514, ENA_T5445822 and ENA_T5603164) were independently confirmed with similar profiles. ENA_T5382514 and ENA_T5445822 increased reporter expression compared to control condition (DMSO) with the same efficiency as the reference agonist shown in **Figure 5A.** Conversely the ENA_T5603164 treatment reduced reporter expression by 45% compared to DMSO condition (**Fig. 5B**) confirming an antagonist profile determined by the initial screening. The secondary analysis was designed in order to qualify the specificity of molecules initially identified. As many potential nuclear receptor ligands are susceptible to have off-target effects on xenobiotic nuclear receptor (CAR and PXR), it was necessary to determine the impact of the identified molecules on these receptors. This specificity screening was processed using mammalian single hybrid system with a Gal4 fused construct. Indeed, contrary to REV-ERBα which interacts only with the corepressor N-CoR, both CAR and PXR are able to recruit several endogenous coactivators. Thus, the use of a single hybrid system (without co-modulator overexpression) allows the assay of ligand activity for CAR and PXR. Both REV-ERBα agonists (ENA_T5382514, ENA_T5445822) were also able to activate human PXR (**Fig. 5C**) and therefore their selectivity is limited. In addition, ENA_T5445822 seemed to be an activator of human hCAR, conversely to ENA_T5603164 which yielded an antagonist profile against this receptor.

## DISCUSSION

The nuclear hormone receptor REV-ERBα plays an important role in the regulation of circadian rhythms of metabolic pathways [25,26]. Significant advances have been made recently that aid our understanding of how ligands can regulate the nuclear receptor REV-ERBα, which was once considered as an orphan receptor [19]. Several studies have led to the identification of novel natural (heme) and synthetic (GSK4112, SR9009, SR9011) ligands for REV-ERBα that modulate REV-ERBα activity *in vitro* with consequent circadian rhythm delay [13], confirming the potential of such ligands to reset the circadian clock after jetlag or shift work [27]. As REV-ERBα is involved in lipid and glucose metabolism, adipogenesis, and vascular wall physiology, it also represents a promising target for the treatment of metabolic abnormalities resulting from chronic aberrant circadian rhythm [26,27]. In order to identify further agonists for REV-ERBα, a mammalian cell-based two-hybrid assay based on the Dual-Glo^®^ Luciferase System was developed, validated, and subsequently employed in a HTS campaign to screen a subset of the Library of Pharmacologically Active Compounds (LOPAC^®1280^) as well as 29568 diverse compounds from Enamine to identify compounds that have the potential to be therapeutics [28].

The development of the mammalian REV-ERBα cell-based two-hybrid assay was a significant challenge as it involved optimization of multiple parameters in an iterative manner, which was particularly time-consuming. After successful optimization of several parameters including the jetPEI/DNA transfection reagent ratio, incubation time for the jetPEI/DNA complex formation, cell density, FBS concentration, signal window, and DMSO tolerance in an assay volume of 20 μl in 384-well plate format with an overall assay time of 24 h. Subsequent to this, the assay was validated using the synthetic ligand GSK4112 but also SR9009 and SR011 to ensure the pharmacology of the assay was consistent with values reported in the literature. The assay was shown to be particularly sensitive with respect to starvation that made use of 1% DCC FBS during the assay. The stimulation time by GSK4112 was shown to be optimal at 16 h, yielding 5.5 μM EC_50_. These results are comparable to those reported in the literature [13]. The Dual-Glo^®^ Luciferase System for measuring REV-ERBα activity was particularly useful as it enabled the identification of a cytotoxic effect for SR9011, in which a decrease in the Renilla signal was observed with increasing compound concentration, which excluded this compound for use as a genuine REV-ERBα agonist.

Subsequent to the development of the mammalian REV-ERBα cell-based two-hybrid assay, screening campaigns against a subset of the LOPAC^®1280^ library and 29568 compounds from a diverse compound library were successfully accomplished. The hits from each screen were profiled in a panel of counter and selectivity assays. These results confirmed that REV-ERBα activity can be modulated pharmacologically and that chemical scaffolds have been identified for optimization. A significant amount of experimental work was performed so as to optimize the large scale screen and it appears that upon scale-up of the assay, although every effort was made so as to ensure the timings for each of the critical parts of the assay were kept constant, there does appear to be a limit beyond which the assay fails to retain its quality. Therefore, the screen was performed by running it in batches of single digit plates per run. A general trend that we found was the gradual deterioration of assay quality as the number of plates being screened were increased. This observation could be due to a number of reasons including sensitivity to temperature and CO_2_, incubator artefacts, liquid handling errors and interference from compounds and any similar assay that is developed should be appropriately validated prior to embarking on a screening campaign [29,30].

The LOPAC^®1280^ screen led to the identification of a small number of hits; amongst them, chelidamic acid (an L-glutamic decarboxylase inhibitor) showed activity in a concentration-dependent manner, yielding an EC_50_ value of 0.36 μM. Furthermore, no increase of firefly luciferase expression could be detected in the transfected cells with an empty vector (Gal4) instead of REV-ERBα plasmid (control transfection). The absence of signal increase in the control transfected cells indicated that chelidamic acid binds specifically to the LBD site of REV-ERBα leading to increased interaction between the co-expressed proteins REV-ERBα and NCoR. Moreover, the compound showed higher activity than the previously known agonist, GSK4112. The larger screen led to the identification of 63 hits of which 48 were confirmed in dose-response studies to be REV-ERBα agonists. The confirmed hits were evaluated in secondary assays. Therefore, these scaffolds can be deemed as chemical starting points for REV-ERBα agonist drug discovery. The hit rate of the assay is acceptable and a number of compounds that were shown to be active against other nuclear receptors adds confidence that the assay is capable of identifying agonists of this important drug target class. This high throughput *in vitro* screening approach complements in silico and low throughput *in vitro* approaches such as ^19^F-NMR that have previously been employed to identify REV-ERBα modulators [31].
